# Impact of early detection and vaccination strategy in COVID-19 eradication program in Jakarta, Indonesia

**DOI:** 10.1186/s13104-021-05540-9

**Published:** 2021-04-12

**Authors:** Dipo Aldila, Brenda M. Samiadji, Gracia M. Simorangkir, Sarbaz H. A. Khosnaw, Muhammad Shahzad

**Affiliations:** 1 Department of Mathematics, Universitas Indonesia, Depok, Indonesia; 2 Department of Mathematics, University of Raparin, Ranya, Kurdistan Region Iraq; 3 Department of Pure and Applied Mathematics, University of Haripur, KP, Pakistan

**Keywords:** COVID-19, Contact tracing, Rapid testing, Vaccination, Basic reproduction number

## Abstract

****Objective**:**

Several essential factors have played a crucial role in the spreading mechanism of COVID-19 (Coronavirus disease 2019) in the human population. These factors include undetected cases, asymptomatic cases, and several non-pharmaceutical interventions. Because of the rapid spread of COVID-19 worldwide, understanding the significance of these factors is crucial in determining whether COVID-19 will be eradicated or persist in the population. Hence, in this study, we establish a new mathematical model to predict the spread of COVID-19 considering mentioned factors.

****Results**:**

Infection detection and vaccination have the potential to eradicate COVID-19 from Jakarta. From the sensitivity analysis, we find that rapid testing is crucial in reducing the basic reproduction number when COVID-19 is endemic in the population rather than contact trace. Furthermore, our results indicate that a vaccination strategy has the potential to relax social distancing rules, while maintaining the basic reproduction number at the minimum possible, and also eradicate COVID-19 from the population with a higher vaccination rate. In conclusion, our model proposed a mathematical model that can be used by Jakarta’s government to relax social distancing policy by relying on future COVID-19 vaccine potential.

## Introduction

At the end of 2019, a novel coronavirus disease was found in Wuhan, China. This disease, commonly known as COVID-19, is caused by the well-known Severe Acute Respiratory Syndrome Coronavirus 2 [1]. The World Health Organization first declared COVID-19 as a threat to the international community on January 30, 2020, and then as a pandemic on March 11, 2020 [2, 3]. As of November 26, 2020, more than 60 million people have been infected by COVID-19, with approximately 1.4 million deaths. The top five countries with COVID-19 incidences, in order, are the USA, India, Brazil, Russia, and France [4]. The debate whether this virus originated from bats continues [5].

COVID-19 spreads among the human population through human-to-human transmission, direct contact with an infected individual, touching a surface that contains the virus, and inhaling free droplets produced by infected individuals [6, 7]. The development and approval of a vaccine for COVID-19 to protect susceptible individuals remains in progress in several countries. Hence, at this time, the most popular intervention to prevent the rapid spread of COVID-19 is through non-pharmaceutical interventions including face masks, social distancing, rapid testing, contact tracing, quarantining, and maintaining individual hygiene [8–11].

Numerous researchers from diverse disciplines have contributed to the study of COVID-19, from the development of a medicines, understanding the spreading mechanism, and forecasting the possible outcome if specific interventions are implemented [8, 9, 12–14]. Recently, many countries have been starting the intervention of vaccination to control the spread of COVID-19. To predict this scenario, several authors have been introduced their model to answer this problem, such as [15, 16]. Indonesia first implemented the COVID-19 vaccine massively in January in Jakarta, with the initial target being health workers and public servants. Motivated from the literature above, we wish to analyze the influence of two existing scenarios in the city of Jakarta, namely the contact tracing and rapid testing, and one new intervention, a vaccination strategy. We construct the proposed model using a well-known compartmental model as a system of nonlinear ordinary differential equations. We then analyze the qualitative behavior of the proposed model regarding the equilibrium and stability criteria and how the basic reproduction number can be used as an indicator to determine whether the disease is eradicated or persists in the population. Using the best-fit parameter to the proposed model with incidence data from the city of Jakarta, Indonesia, we show how far we can rely on vaccination strategy to relax the social distancing policy or even eradicate COVID-19 spread from the population.

## Main text

### Methods

Virtually all countries in the world are eagerly awaiting the availability of an effective vaccine to combat the spread of COVID-19. A vaccine must fulfill at least two requirements before it can be widely used in humans. The first is that the vaccine must be safe for all population types (age, sex, race). The second is that the vaccine must demonstrate acceptable efficacy against the pathogen. There are numerous challenging aspects to the implementation of a COVID-19 vaccine; one is the magnitude of the intervention required to address a sufficient population such that herd immunity in the population can be achieved. To answer this question, we must analyze the impact of a vaccination before it is actually implemented in the field. One approach is the use of a mathematical model to predict the effect of the vaccine on the population.

**Fig. 1:**
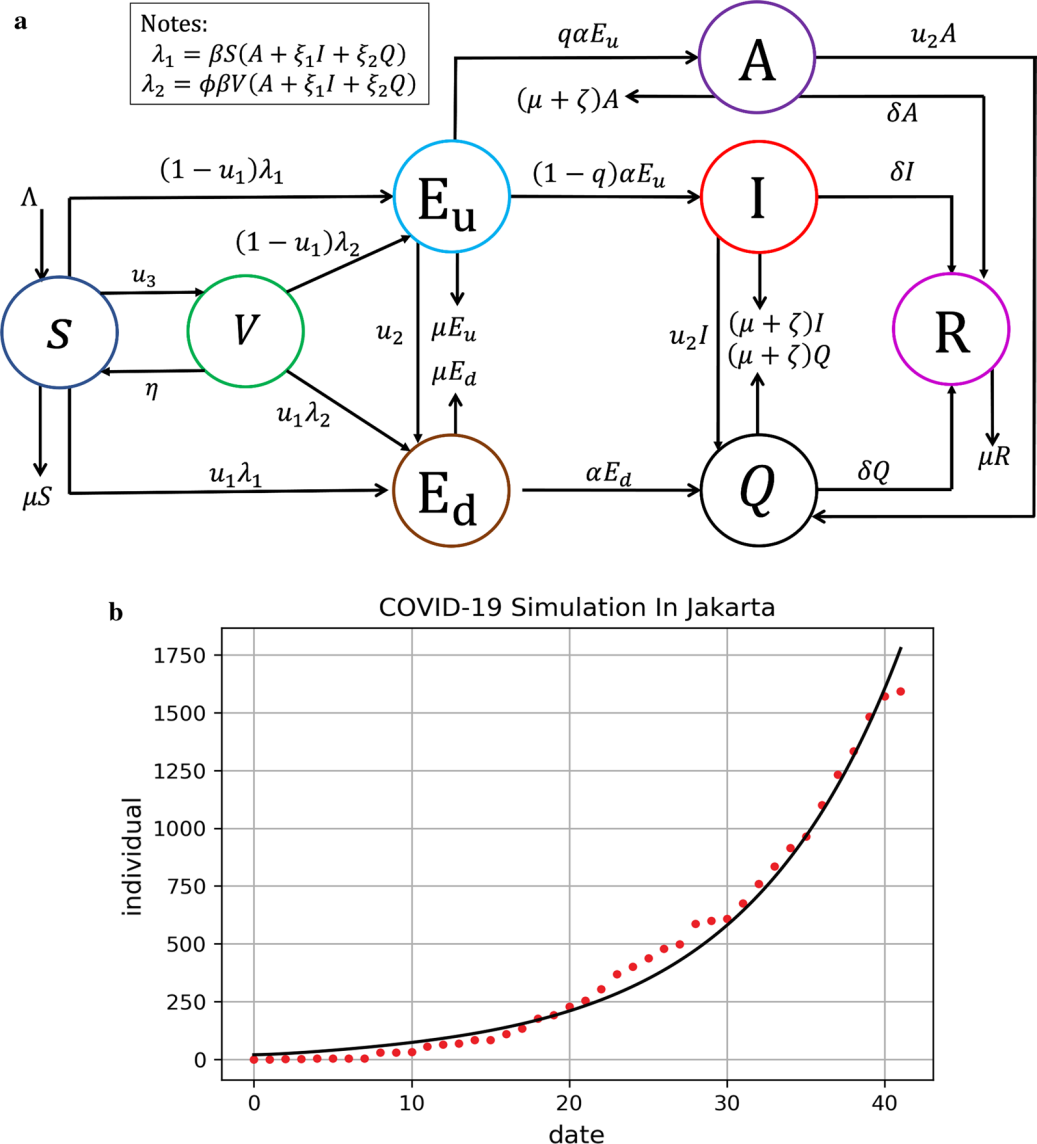
Transmission diagram of COVID-19 model in System 1**a** and data fitting of quarantined individual in the early spread of COVID-19 in Jakarta (March 03 to May 20, 2020) **b**

A flow chart of the dynamics of the proposed model is shown in Fig. 1a. We consider several important factors including detected and undetected cases, contact tracing and rapid testing to find undetected cases, quarantining to avoid further possible infection from the detected infected individuals, and vaccination strategy to minimize successful contact probability. To accommodate these factors, we divide our population based on their health status as follows: individuals who are vulnerable to COVID-19 (*S*), vaccinated individuals (*V*), detected and undetected exposed individuals ($E_u$ and $E_d$, respectively), asymptomatic and symptomatic individuals (*A* and *I*, respectively), quarantined (*Q*) and recovered individuals (*R*).

Based on the aforementioned explanation, the mathematical model for COVID-19 spread, considering contact tracing, rapid testing, symptomatic/asymptomatic cases, and quarantine intervention, is expressed by the following system of ordinary differential equations.

1 $$\begin{aligned} \frac{dS}{dt} & =  \Lambda - \beta S \left( A + \xi _1 I + \xi _2 Q\right) - \mu S - u_3 S + \eta V, \\ \frac{dV}{dt} &=  u_3 S - \eta V - \phi \beta V \left( A + \xi _1 I + \xi _2 Q\right) - \mu V,  \\ \frac{dE_u}{dt} &=  (1-u_1)\beta (S+\phi V) \left( A + \xi _1 I + \xi _2 Q\right) - \alpha E_u - \mu E_u - u_2 E_u,  \\ \frac{dE_d}{dt} &= u_1 \beta (S+\phi V) \left( A + \xi _1 I + \xi _2 Q\right) - \alpha E_d - \mu E_d + u_2 E_u ,  \\ \frac{dA}{dt} &=  q \alpha E_u - \delta A - u_2 A - (\mu +\zeta )A,  \\ \frac{dI}{dt} &=  (1-q)\alpha E_u - \delta I - u_2 I - (\mu +\zeta )I,  \\ \frac{dQ}{dt} & =  \alpha E_d + u_2 (A+I) - \delta Q - (\mu +\zeta )Q, \\ \frac{dR}{dt} &=  \delta (A+I+Q) - \mu R, \end{aligned}$$

Description of all parameters given in Table 1. Parameter were estimated using the incidence data in Jakarta in the early spreading period of COVID-19, and the result is given in 1(b). In the above model, we assume that all newborns ($\Lambda$) are healthy. Vaccination is given only to *S* with a rate of $u_3$ which will transfer them into *V*. It is assumed that the vaccine has a validity period of $\eta ^{-1}$ and does not protect people perfectly from COVID-19. Hence, vaccinated individual may get infected by COVID-19, but the transmission rate $\beta$ were reduced by $\phi$. Due to contact trace $u_1$, new infected individual separated into detected $(E_d)$ and undetected $(E_u)$ class. The undetected case $E_d, A$ and *I* can be traced by the rapid/swab test intervention with the rate of $u_3$. Our model assumes that the detected cases from *A* and *I* will be forced to conduct quarantine until they get recovered from COVID-19.

**Table 1:** Description of model parameters in system 1

Par.	Description	Baseline value	Ref.
$\Lambda$	Recruitment rate	$\frac{10\,467\,629}{65 \times 265}$	[8]
$\mu$	Natural death rate	$\frac{1}{65 \times 365}$	[8]
$\beta$	Transmission rate	$4.74396 \times 10^{-8}$	Fitted
$\xi _1$	Modification parameter to reduce infectiousness of *I*	0.9	Fitted
$\xi _2$	Modification parameter to reduce infectiousness of *Q*	0.3	Fitted
$\alpha$	Transition to infected compartment owing to incubation period	$\frac{1}{5.1}$	[17, 18]
$\delta$	Recovery rate	0.1	[17]
*q*	Proportion of exposed individuals $E_u$ become asymptomatic *A*	0.6	[19, 20]
$\zeta$	Death rate due to COVID-19	0.06	[12]
$u_1$	Contact trace intervention	0.5	Fitted
$u_2$	Rate of rapid testing	0.083	[8]
$u_3$	Vaccination rate	Varrying	Assumption
$\eta$	Duration of vaccine still valid	Varrying	Assumption
$\phi$	Vaccine efficacy	0.8	Assumption

### COVID-19 free equilibrium and the basic reproduction number

Model 1 has one trivial equilibrium, namely the COVID-19 free equilibrium, which is given by

2 $$\begin{aligned} E_0 &=   \left( S, V, E_u, E_d, A, I, Q, R\right)  \\ & = \left( \frac{\Lambda }{\mu } \times \frac{\eta + \mu }{\eta + \mu + u_3 }, \frac{\Lambda }{\mu } \times \frac{u_3}{\eta + \mu + u_3 } 0,0,0,0,0,0\right) . \end{aligned}$$

The total population at $E_0$ is $\frac{\Lambda }{\mu }$. In Model 1, the susceptible population is separated into non-vaccinated and vaccinated populations. The ratio is given by

3 $$\begin{aligned} \rho = \frac{V}{S}|_{E_0} = \frac{u_3}{\eta + \mu }. \end{aligned}$$

It can be observed that this ratio is directly proportional to the rate of vaccination $u_3$ and inversely proportional to the vaccination period $\eta$. Hence, except only relying on massive vaccination rate on to the population, the policy maker also should consider the vaccine duration that will be used in the field.

Next we calculate the basic reproduction number of system (1). Let us define the transition (*F*) and transmission (*V*) matrix of system (1) evaluated at $E_0$ as follows.

$$\begin{aligned} F= & {} \left[ \begin{array}{ccccc} 0&{}0&{} \left( 1-u_{{1}} \right) \beta \, \left( \phi \,V+S \right) &{} \left( 1-u_{{1}} \right) \beta \,\xi _{{1}} \left( \phi \,V+S \right) &{} \left( 1-u_{{1}} \right) \beta \,\xi _{{2}} \left( \phi \,V+S \right) \\ 0&{}0&{}\beta \,u_{{1}} \left( \phi \,V+S \right) &{}\beta \,u_{{1}}\xi _{{1}} \left( \phi \,V+S \right) &{}\beta \,u_{{1}}\xi _{{2}} \left( \phi \,V+S \right) \\ 0&{}0&{}0&{}0&{}0\\ 0&{}0&{}0&{}0&{}0 \\ 0&{}0&{}0&{}0&{}0\end{array} \right] , \nonumber \\ V= & {} \left[ \begin{array}{ccccc} -\alpha -\mu -u_{{2}}&{}0&{}0&{}0&{}0 \\ u_{{2}}&{}-\alpha -\mu &{}0&{}0&{}0\\ \alpha \,q&{}0&{}-\delta -\mu -\zeta -u_{{2}}&{}0&{}0\\ \left( 1-q \right) \alpha &{}0&{}0&{}-\delta -\mu -\zeta -u_{{2}}&{}0 \\ 0&{}\alpha &{}u_{{2}}&{}u_{{2}}&{}-\delta -\mu -\zeta \end{array} \right] . \end{aligned}$$

Since we have three zero rows on *F*, the next generation matrix of system (1) is taken by the spectral radius of $NGM = -E^TFV^{-1}E,$ where $$E=\left[ \begin{array}{ccccc} 1&{}0&{}0&{}0&{}0\\ 0&{}1&{}0&{}0&{}0 \end{array} \right] ^T$$. Therefore, the basic reproduction number of system (1) is given by

4 $$\begin{aligned} {\mathcal {R}}_0 = {\mathcal {R}}_0^{'} \frac{\phi u_3 + \eta + \mu }{u_3 + \eta + \mu }, \end{aligned}$$

and ${\mathcal {R}}_0^{'} = (1-u_1) \beta B + u_1 {\frac{\Lambda \,\beta \,\xi _{{2}} \alpha }{\mu \, \left( \alpha +\mu \right) \left( \mu +\delta +\zeta . \right) }},$ with $B = {\frac{q\,\alpha \,\Lambda }{\mu \, \left( \mu +\delta +\zeta +u_{{2} } \right) \left( \alpha +\mu +u_{{2}} \right) }}+{\frac{\,\Lambda \,\xi _{{1}} \left( 1-q \right) \alpha }{\mu \, \left( \mu +\delta +\zeta + u_{{2}} \right) \left( \alpha +\mu +u_{{2}} \right) }} + {\frac{\, \Lambda \,\xi _{{2}}\alpha \,u_{{2}} \left( \delta +2\,\mu +\zeta +u_{{2}}+ \alpha \right) }{\mu \, \left( \alpha +\mu \right) \left( \alpha +\mu +u_ {{2}} \right) \left( \mu +\delta +\zeta +u_{{2}} \right) \left( \mu +. \delta +\zeta \right) }}.$ The following results follows from Theorem 2 in [21]

#### **Theorem 1**

The COVID-19 free equilibrium point of model 1 is locally asymptotically stable if ${\mathcal {R}}_0<1$, and unstable if ${\mathcal {R}}_0>1$.

Please note that ${\mathcal {R}}_0^{'}$ is the basic reproduction number of system (1) where no vaccine intervention implemented. From the expression of ${\mathcal {R}}_0^{'}$, it can be observed that ${\mathcal {R}}_0 < {\mathcal {R}}_0^{'}$ because $K:=\frac{\phi u_3 + \eta + \mu }{u_3 + \eta + \mu }<1$. The higher the quality of the vaccine (smaller value of $\phi$), the smaller is the value of *K*. The parameter *K* represents the effect of vaccine implementation in reducing the initial basic reproduction number, which depends on the rate of vaccination and quality of the vaccine. Hence, it can be concluded that the implementation of a vaccination at a constant rate $u_3$ reduces the basic reproduction number by *K* percent.

## Results

According to Theorem 1, there is a chance that COVID-19 could be eradicated from the community if ${\mathcal {R}}_0$ is less than unity. Because the partial derivative of ${\mathcal {R}}_0$ with respect to the contact tracing parameter $u_1$ given by

$$\begin{aligned} \frac{\partial {\mathcal {R}}_0}{\partial u_1} = -\frac{\alpha \beta \Lambda ((\xi _1-\xi _2)+q(1-\xi _1)) K}{\mu (\mu + \zeta + u_2 + \delta )(\alpha + \mu + u_2)} \end{aligned}$$

is always negative, we can conclude that increasing the intensity of contact tracing in the community can reduce the value of the basic reproduction number. Hence, there is an acceptable probability that COVID-19 could be eradicated from the community if the contact tracing effort is increase. From it can be stated that the total number of infected individuals (exposed, asymptomatic, symptomatic, and quarantine) is inversely proportional to contact tracing intervention. It can be observed that with more intense contact tracing in the field, the outbreak could be reduced significantly, and the time of the occurrence of the outbreak could be delayed for a significant period.

The results regarding the rapid testing intervention are as follows. Because

$$\begin{aligned} \frac{\partial {\mathcal {R}}_0}{\partial u_2} = -{\frac{\alpha \,\Lambda \,\beta \, \left( 1- u_{{1}} \right) ((\xi _1-\xi _2)+q(1-\xi _1)) \left( \alpha +\delta +2\,\mu +\zeta +2\,u_{{2}} \right) K}{ \left( \alpha +\mu +u_{{2}} \right) ^{2} \left( \delta +\mu +\zeta +u_{{2}} \right) ^{2}\mu }}\end{aligned}$$

is always negative, we can observe that increasing the intensity of rapid testing to identify the undetected cases in the field and force them to conduct self-isolation could increase the probability of eradicating COVID-19 from the population. It is evident from the dynamics of the total infected individuals, that the intervention of rapid testing demonstrated similar qualitative results. However, please note that rapid testing is more sensitive in reducing the number of infected individuals compared to the intervention of contact tracing. To increase the positive results of the rapid testing in the population, not only should the number of tests be increased but also rapid testing sampling must be conducted immediately on the target.

Furthermore, we can observe that

$$\begin{aligned} \frac{\partial {\mathcal {R}}_0}{\partial \beta } = (1-u_1)B + u_1 {\frac{\Lambda \,\xi _{{2}} \alpha K}{\mu \, \left( \alpha +\mu \right) \left( \mu +\delta +\zeta \right) }}\end{aligned}$$

is always positive, which indicates that reducing the success transmission rate could reduce the basic reproduction number linearly. This parameter depends on the duration and intensity of the contact, and the ease of the virus spread through susceptible individuals from infected individuals. Hence, to reduce secondary infection, it is necessary to reduce the contact rate. This can be accomplished through massive social distancing or even conducting lockdowns in endemic areas. However, this intervention comes at a high cost, not only from an epidemiological point of view but also from the social and economic points of view. As an alternative, reducing the infection rate can be achieved by forcing the population to use face masks whenever they interact socially with others. This intervention is easier to implement because every individual has their own responsibility to cut the chain of COVID-19 transmission, starting with themselves.

**Fig. 2:**
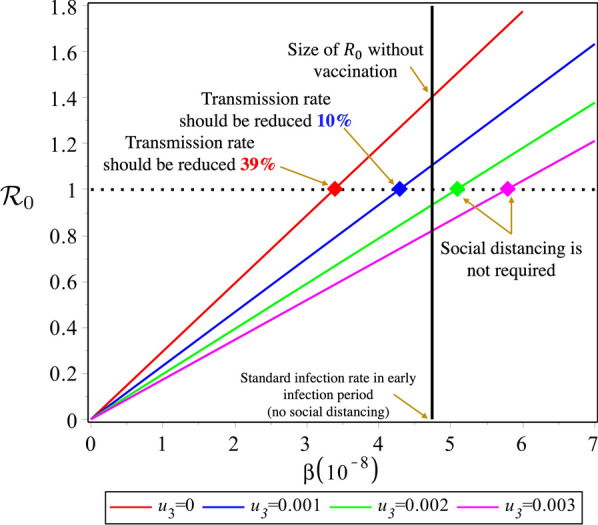
Effect of vaccine on sensitivity of infection rate to basic reproduction number

In a similar way, we also can analyze the impact of vaccination to ${\mathcal {R}}_0$. Taking the derivative of ${\mathcal {R}}_0$ respect to $u_3$ gives

$$\begin{aligned} \frac{\partial {\mathcal {R}}_0}{\partial u_3} = {\mathcal {R}}_0^{'} \frac{(1-\phi u_3)+(1-\phi )(\xi +\mu )}{(u_3+\eta +\mu )^2} \end{aligned}$$

which always negative. Hence, vaccination rate is inversely proportional to ${\mathcal {R}}_0$. From Fig. 2, it can be observed by the red curve that without implementation of a vaccine and social distancing, the basic reproduction number is greater than unity, which indicates a high chance of COVID-19 being endemic in the population. Furthermore, we can observe that without vaccination intervention, the transmission rate $\beta$ must be reduced by greater than $39\%$ to maintain the basic reproduction number less than unity, and thus provide an opportunity to eradicate COVID-19 from the population. With the implementation of the vaccine at a constant rate of 0.001, we can see that the transmission rate must only be reduced by 10%, rather than 39%. This means that it is not necessary to over-implement social distancing; hence, economic and social activities could function more normally. If the vaccination rate is given at a higher rate, which in our experiment we chose 0.002 and 0.003, it can be observed that social distancing is no longer required in that the basic reproduction number decreases to less than unity. Hence, we can conclude that the intervention of vaccination has an excellent potential to allow the government to relax the social distancing intervention and to eradicate COVID-19 from the population.

### Discussion

Using the best-fir parameter based on incidence data in Jakarta, our proposed model indicates that the control measures of early detection, rapid test and vaccination has a potential to avoid huge size of endemic of COVID-19 in the city of Jakarta. Implementation of vaccination strategy in the near future can be used to relaxed social distancing policy such that the social and economy activity in the population can back to normal, and keep the spread of COVID-19 as low as possible.

## Limitations

Our results in this article provide preliminary results in our effort to understand how far we can rely on a potential COVID-19 vaccine, especially in Jakarta. Important questions remain undetermined in this research, and the precise answers must be found as soon as possible. The important questions that should be considered in future research are as follows: 1) what type of population should be considered for the vaccine intervention? This is important because the budget for implementation is frequently limited, and 2) although our result demonstrate a potential positive effect of the vaccine for eradicating COVID-19, how far can we rely on this intervention? How high a vaccine efficacy is required to impact the level of success of the COVID-19 eradication program? These questions not yet answered on our model.

## Data Availability

The data used to support findings of this study is available from the corresponding author upon request.

## Abbreviation

COVID-19: Coronavirus disease 2019
